# Sirolimus: A Successful Medical Treatment for Head and Neck Lymphatic Malformations

**DOI:** 10.1155/2019/2076798

**Published:** 2019-03-24

**Authors:** Steven Curry, Andrew Logeman, Dwight Jones

**Affiliations:** Department of Otolaryngology-Head and Neck Surgery, University of Nebraska Medical Center, Omaha, Nebraska, USA

## Abstract

Lymphatic malformations are abnormalities that arise in the developing lymphatic system, most frequently presenting in the head and neck. They are typically treated with sclerotherapy, laser therapy, or surgery for localized lesions. Sirolimus, an inhibitor of the mammalian target of rapamycin, is a relatively new medical therapy for the treatment of vascular malformations. This case report presents the improvements and complications seen in a female infant who was diagnosed with a large lymphatic malformation on prenatal ultrasound and has been treated with sirolimus during the first 9 months of life.

## 1. Introduction

Lymphatic malformations (LMs) are slow-flow cystic anomalies that develop from abnormal embryologic formation of the developing lymphatic system, which enlarge by expansion of the lesion in contrast to proliferation which occurs in vascular tumors. LMs present most frequently in the head and neck and are usually present at birth or by two years of age. Upregulation of the PI3K/Akt/mTOR pathway may be a causal factor in the development of these abnormal lymphatic vessels. Sirolimus, which inhibits the mammalian target of rapamycin (mTOR) to decrease cell proliferation and lymphangiogenesis, is a relatively new medical therapy for vascular malformations [1, 2].

## 2. Case Presentation

**Figure 1:**
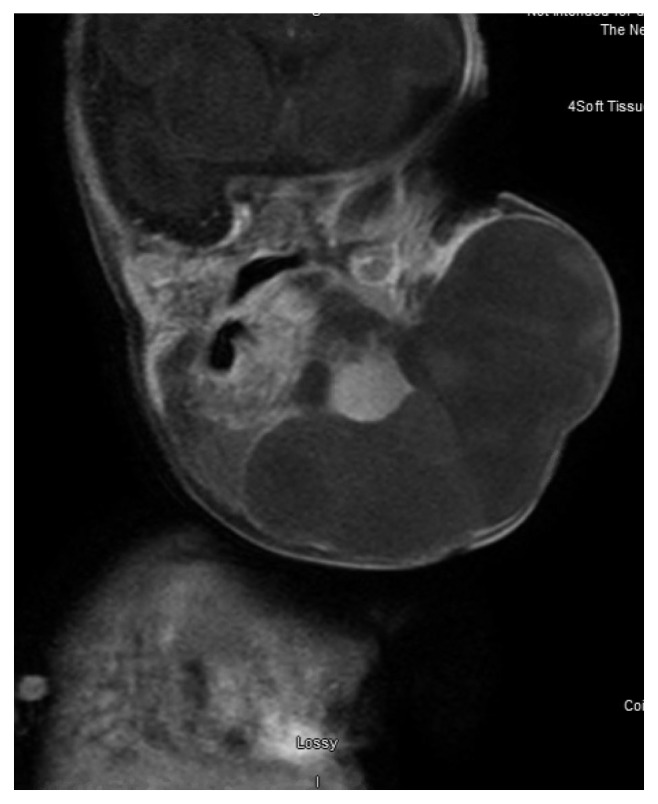
T1+ contrast coronal MRI demonstrates a large mixed-cystic LM extending from the left neck into the chest with recent hemorrhage, measuring 8.1 × 5.7 × 5.5 cm.

**Figure 2:**
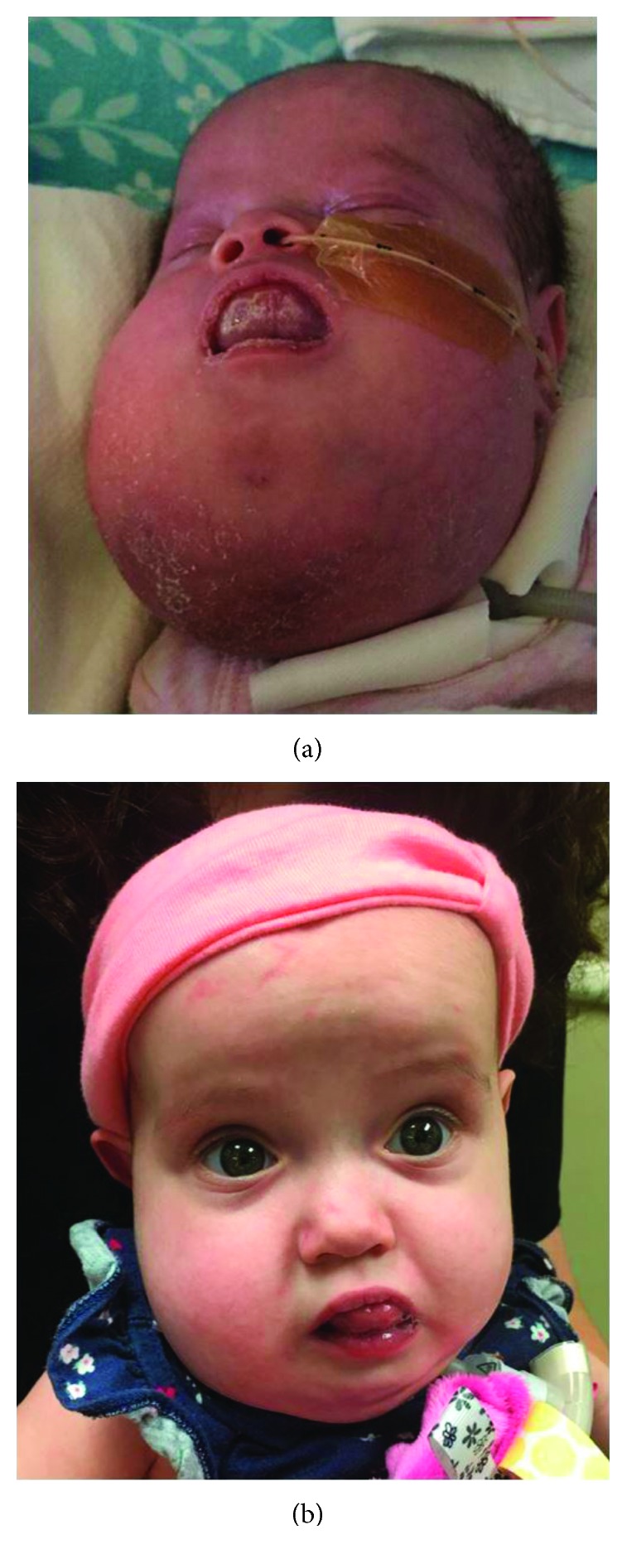
Prior to sirolimus treatment, there is significant mass effect involving the tongue, floor of mouth, and neck. After eight months of sirolimus treatment, the patient is able to close her mouth around her tongue and has markedly decreased mass effect on the face, mouth, and neck.

A prenatal ultrasound revealed a large LM. The mother went into preterm labor at 31 weeks and 6 days gestational age and delivered via c-section in association with planned exit procedure to stabilize the airway. MRI of the neck on day of life (DOL) 3 showed a very large multiloculated and multispatial mass (Figure 1) within the left neck extending from the left inferior periauricular region through the upper chest with extension across the midline (de Serres stage V). The lesion demonstrated hemorrhage fluid levels and associated mass effect most compatible with a large mixed-cystic LM with recent hemorrhage and complete collapse of the visualized left lung. It measured approximately 8.1 × 5.7 × 5.5 cm. She underwent ultrasound-guided drain placement DOL 10 and sclerotherapy with doxycycline on DOL 11. The drain was then removed on DOL 16. She underwent tracheostomy on DOL 17 and required respiratory support for a total of 56 days before weaning to the trach collar (Figure 2(a)). The patient was started on sirolimus therapy at a dose of 0.8 mg/m^2^ on DOL 22. Sirolimus therapy has since been maintained at a target serum trough level of 10 to 15 ng/mL. She was initially fed through an orogastric tube, followed by a nasogastric tube, and then through a gastrostomy tube once placed on DOL 52. The patient developed peripheral edema and hypertension thought to be due to sirolimus and which were treated with chlorothiazide beginning on DOL 37. She developed cellulitis of the LM on DOL 26 and again at 5 months of age, treated each time with IV antibiotics. Since discharge from the hospital, the patient has been followed in the otolaryngology clinic at 3-month intervals. The patient has now been on sirolimus treatment for 8 months with reduction in the bulk of the LM. Her functional improvement includes now being able to close her mouth around her tongue (Figure 2(b)).

## 3. Discussion

Treatment of LMs has typically been managed with sclerotherapy, laser therapy, or surgery for localized lesions. Diffuse malformations present significant challenges due to the morbidity of interventional treatments to achieve local control. There are currently no guidelines for treatment of LMs [3]. mTOR inhibitors, such as sirolimus, have recently been used for their antilymphangiogenic properties in treating LMs [4]. A recent phase II trial showed that sirolimus is efficacious and well tolerated in patients with complicated vascular anomalies [1].

This case presents the clinical improvements in an infant female over the first 9 months of life who has been treated with sirolimus beginning in the first month of life following drainage and doxycycline sclerotherapy for a large LM of the left neck and chest.

As an immunosuppressant, sirolimus can cause neutropenia and may predispose the patient to an increased risk of infection. Furthermore, lymphatic malformations are known to become infected and hemorrhage. It has been reported, however, that hemorrhage and infections in LMs may be less frequent and milder after treatment with sirolimus [1]. Additionally, sirolimus may be more effective in the treatment of LMs in younger patients [4]. In the present case, treatment with sirolimus has been effective and well tolerated in the neonatal and infant periods. This case highlights the successful medical management of a large, complex LM, while avoiding the morbidity of surgery. Medical therapies that target other elements in the PI3K/Akt/mTOR pathway are currently being studied, including PI3K inhibitors, and they may be promising treatments in the future.

This report was reviewed and determined to be exempt by the University of Nebraska Medical Center Institutional Review Board.

## References

[1] Adams D. M., Trenor C. C., Hammill A. M. (2016). Efficacy and safety of sirolimus in the treatment of complicated vascular anomalies. *Pediatrics*.

[2] Hammill A. M., Wentzel M., Gupta A. (2011). Sirolimus for the treatment of complicated vascular anomalies in children. *Pediatric Blood & Cancer*.

[3] Maruani A., Boccara O., Bessis D. (2018). Treatment of voluminous and complicated superficial slow-flow vascular malformations with sirolimus (PERFORMUS): protocol for a multicenter phase 2 trial with a randomized observational-phase design. *Trials*.

[4] Strychowsky J. E., Rahbar R., O’Hare M. J., Irace A. L., Padua H., Trenor C. C. (2018). Sirolimus as treatment for 19 patients with refractory cervicofacial lymphatic malformation. *Laryngoscope*.

